# Tyrosine-Modification of Polypropylenimine (PPI) and Polyethylenimine (PEI) Strongly Improves Efficacy of siRNA-Mediated Gene Knockdown

**DOI:** 10.3390/nano10091809

**Published:** 2020-09-10

**Authors:** Sandra Noske, Michael Karimov, Achim Aigner, Alexander Ewe

**Affiliations:** Rudolf-Boehm-Institute for Pharmacology and Toxicology, Clinical Pharmacology, Leipzig University, Faculty of Medicine, 04107 Leipzig, Germany; Sandra.noske@medizin.uni-leipzig.de (S.N.); michael.karimov@medizin.uni-leipzig.de (M.K.)

**Keywords:** polypropylenimine dendrimers, polyethylenimines, tyrosine-modified polypropylene-imines, tyrosine-modified polyethlyenimines, PPI-Y, siRNA transfection, polymeric nanoparticles

## Abstract

The delivery of small interfering RNAs (siRNA) is an efficient method for gene silencing through the induction of RNA interference (RNAi). It critically relies, however, on efficient vehicles for siRNA formulation, for transfection in vitro as well as for their potential use in vivo. While polyethylenimines (PEIs) are among the most studied cationic polymers for nucleic acid delivery including small RNA molecules, polypropylenimines (PPIs) have been explored to a lesser extent. Previous studies have shown the benefit of the modification of small PEIs by tyrosine grafting which are featured in this paper. Additionally, we have now extended this approach towards PPIs, presenting tyrosine-modified PPIs (named PPI-Y) for the first time. In this study, we describe the marked improvement of PPI upon its tyrosine modification, leading to enhanced siRNA complexation, complex stability, siRNA delivery, knockdown efficacy and biocompatibility. Results of PPI-Y/siRNA complexes are also compared with data based on tyrosine-modified linear or branched PEIs (LPxY or PxY). Taken together, this establishes tyrosine-modified PPIs or PEIs as particularly promising polymeric systems for siRNA formulation and delivery.

## 1. Introduction

Severe diseases including cancer are often associated with the aberrant or uncontrolled expression of pathologically relevant genes. The use of small interfering RNAs (siRNAs) for inducing gene knockdown through RNA interference (RNAi) has become a powerful and promising technology to specifically downregulate target genes by harnessing a highly conserved cellular protein machinery called RNA-induced silencing complex (RISC) [1,2]. Today, siRNAs for any target RNA sequence of interest can be easily designed and synthetically generated [3]. They are thus also able to target proteins which are otherwise undruggable by small molecules and offer new therapeutic strategies. This also includes the knockdown of non-protein encoding mRNAs such as “long noncoding RNAs” (lncRNA) which are crucial biological regulators [4]. However, siRNAs are large and negatively charged molecules, which impair their cellular delivery and uptake, and are very susceptible to nuclease degradation. To address these issues, siRNAs can be chemically modified, e.g., by introducing non-natural nucleotide analogues or the covalent coupling targeting moieties [5,6], and/or it is often required to formulate them into nanoparticles.

In the recent years, several lipid molecules [7,8,9], cationic polymers [10,11], inorganic nanoparticles and other systems have been investigated for nucleic acid delivery [12,13,14]. Cationic materials are able to form nano-sized complexes through electrostatic interactions and efficiently protect their payload. For liver-related diseases, suitable siRNA carriers have already been established and, more recently, the first siRNA-based therapeutics have been translated into the clinic. In 2018, Patisiran (ONPATTRO^®^) was approved by the FDA. Relying on a liposomal siRNA formulation, Patisiran downregulates transthyretin in the liver for the treatment of hereditary transthyretin-mediated amyloidosis (hATTR) [15]. The second siRNA therapeutic, Givosiran (GIVLAARI^®^), was approved one year later for targeting aminolevulinic acid synthase 1 (ALAS1) in the treatment of acute hepatic porphyrias (AHPs). Givosiran is an siRNA bearing extensive chemical modifications and it is coupled to GalNAc for the targeted delivery to hepatocytes [16,17]. Despite these impressive advances, most siRNA delivery systems are unable to efficiently deliver siRNAs into target sites other than the liver, thus showing serious limitations, for example, in anti-tumor therapies [18,19]. In this context, polymeric nanoparticles may be particularly promising due to their versatility based on the implementation of chemical modifications.

There are several rate-limiting steps which need to be addressed when using nanosized carriers for systemic application. On the extracellular level, nanocarriers bind serum-components which may lead to aggregation, decomposition and/or opsonization by phagocytic cells. Poor tissue penetration and low internalization rates, followed by insufficient endo-/lysosomal release and intracellular siRNA release into the correct compartment are further limiting steps on the cellular/intracellular level [20,21].

**Figure 1:**
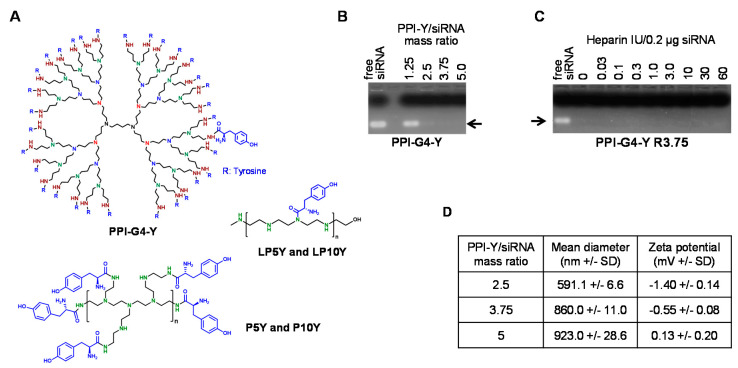
(**A**) Structures of tyrosine-modified PPI-G4 (“PPI-G4-Y”; upper panel) as well as tyrosine-grafted branched or linear polyethylenimines (PEIs) (lower panels; numbers indicate molecular weights of PEI). (**B**) Analysis of PPI-G4-Y-mediated siRNA complexation efficacy, dependent on polymer/siRNA mass ratios. (**C**) Determination of PPI-G4-Y/siRNA complex stability by heparin displacement assay. (**D**) Size and zeta potential of PPI-G4-Y/siRNA complexes at different mass ratios.

Cationic polymers are promising candidates for addressing these issues, and in particular polyethylenimines (PEIs) are widely explored in this regard. PEIs are commercially available in branched and linear structures over a wide range of molecular weights (0.8–100 kDa). Branched PEIs comprise of amines in every third position, at a 1:2:1 ratio of 1°:2°:3° amines. The linear form consists of only secondary and a very few primary amines [22,23,24,25]. Polypropylenimine dendrimers (PPI) are another class of commercially available cationic polymers. PPIs are highly branched molecules with well-defined structure and size. They are composed of a diaminobutane core molecule and propylene imine repeating units. PPI dendrimers contain only tertiary amines in the interior and primary amines at the surface (see Figure 1); the number of repeating units determines the so-called ‘generation’ of the dendrimer and the number of surface functionalities [26]. However, in contrast to (larger) pDNA molecules, PPI dendrimers have not been explored so extensively for delivering siRNAs [27]. Marked differences have been observed between the delivery of siRNA and pDNA when choosing the optimal PPI generation. The smaller G3 PPI dendrimer (1687 Da, 16 1° amines) has been identified as most efficient for pDNA complexation and gene expression [28]. For the delivery of the smaller and more rigid siRNA molecules or small oligonucleotides, the G4 PPI (3514 Da, 32 1° amines) showed the highest uptake rates and gene knockdown efficiencies, when compared G3 and G5 PPI dendrimers [29]. Still, another study used the PPI G3 for the siRNA mediated knockdown of the ubiquitin ligase ITCH in various pancreatic cancer cell lines, showing an ~ 50% downregulation only at high siRNA concentrations (5 µg/200 µL) [30].

When considering PEIs or PPIs for therapeutic nucleic acid delivery, there are major concerns impeding their clinical translation. While it is well known that the transfection efficacy of these polymers increases with the molecular weight or number of generations, this is usually associated in parallel with higher cytotoxicity. Furthermore, PEI and PPI complexes tend to aggregate over time in physiological buffers or upon contact with biological fluids, e.g., serum [28,31,32,33].

Due to the large content of reactive amines in PEIs and PPIs, their chemical modification has been extensively explored, for further improvement of PEI- and PPI-based delivery systems. A general approach towards reducing cytotoxicity of cationic polymers is the shielding of the strong cationic charge by PEGylation. Although this strategy improved the biocompatibility and colloidal stability of the complexes, their cellular uptake and transfection efficacies were decreased [34,35]. To address this issue, targeting moieties have been introduced for cell specific internalization of pDNA- and siRNA complexes. This was shown for the coupling of the luteinizing hormone-releasing hormone (LHRH) peptide or folate onto the distal end of G4 or G5 PPI dendrimers [36,37]. In another approach, G4 PPI was modified with maltose for improved biocompatibility, and the loss of cellular uptake was compensated by coupling of an anti-EGFRvIII single chain antibody [38]. Other chemical modifications focused only on pDNA delivery. Another widely explored method for modifying cationic polymers is the introduction of lipophilic molecules. Here, biological activities were improved; for example after alkylcarboxylation of G4 PPI with 10-bromodeconoic acid or fluorination of G3-G5 PPIs with heptafluorobutyric acid [39,40]. Other studies explored PPI copolymers like PPI-poly-L-lysine, PPI-oligoethylenimine or PPI-Pluronic^®^ P123 [33,41,42]. Smaller molecules were found to be efficient as well. The coupling of amino acids arginine, leucine or lysine strongly enhanced gene expression efficiencies of G3 PPI-based complexes and even of the very small G2 PPI [43,44]. Similarly, the modification of the larger G4/G5 PPIs with heterocyclic amines (histidine, piperazine-2-carboxyilic acid and 3-pyridyl acetic acid) reduced cytotoxicity, while leading to higher gene expression levels [45].

In stark contrast, chemical modifications of PPI-based systems with the aim of improving siRNA delivery have been barely explored at all. For creating a library, G1 PPI was modified with various alkyl epoxides, prior to formulation with PEG-lipids and siRNA to form nanoparticles. The most efficient derivative was a C16-modified G1 PPI that was able to downregulate Tie2 in endothelial cells in vitro and in vivo [46]. Another approach anchored G3 PPI onto gold nanoparticles. The Au-G3/siRNA complexes achieved a 70% knockdown of the target gene BCl-2, with negligible nonspecific toxicity [47]. Alternatively, an optimized formulation rather than a covalent modification employed the polyphenol epigallocatechin-3-O-gallate (EGCG) for pre-mixing with siRNA, prior to complexation with a G2 PPI. This ternary complex reduced luciferase reporter gene activity by 70% [48]. These findings identify PPI dendrimers as attractive candidates for further improvement. Moreover, PPI dendrimers are available at large scales and more defined structures compared to “normal” polymers, which is of particular importance when it comes to medical applications [49].

Recently, we selected a range of branched low molecular weight PEIs (2, 5, and 10 kDa) which were modified with tyrosine at the primary amines, yielding the tyrosine-grafted PEIs P2Y, P5Y and P10Y, respectively. This modification strongly enhanced siRNA-mediated knockdown efficiencies of different target genes in various cell lines at very low polymer/siRNA ratios, with a 70–90% target gene downregulation. Notably, even in the case of the smallest 2 kDa PEI, which is otherwise biologically inactive, tyrosine modification achieved knockdown efficacies of 50%. In addition, the colloidal stability of the tyrosine-modified PEI complexes upon incubation with serum was markedly improved [50,51,52]. More recently, we extended this strategy towards the modification of linear PEIs. Linear PEIs are more biocompatible than their branched counterparts and they can be produced with narrow polydispersities [53]. However, in their unmodified form, linear PEIs cannot sufficiently deliver siRNA into cells. We selected 2.5, 5, 10 and 25 kDa linear PEIs for tyrosine modification. Again, all new polymer derivatives, called LP2.5Y, LP5Y, LP10Y and LP25Y, showed excellent knockdown efficacies of 80–90% in various cell lines, including hard-to-transfect cells. The most active candidates, the linear LP10Y as well as the branched P5Y and P10Y, have also been successfully evaluated in tumor xenograft mouse models for siRNA-mediated oncogene targeting [54].

Thus, as shown previously, and in this paper, the tyrosine modification of branched or linear, small PEIs markedly improves their efficacy and biocompatibility. Based on these findings, we have now extended this approach for the first time towards a PPI dendrimer and present the superior properties of a tyrosine-modified fourth generation PPI (“PPI-G4-Y”). This covers the physicochemical characterization of the PPI-G4-Y/siRNA complexes which show high complexation efficacy and complex stability, and little surface charge. High biological activity and low cytotoxicity were found and compared to various tyrosine-modified branched and linear PEI complexes in different cell lines. In combination with first in vivo biocompatibility studies, our data identify PPI-G4-Y dendrimers as versatile and particularly promising siRNA delivery platform.

## 2. Materials and Methods

### 2.1. Materials

All chemicals and reagents were of analytical grade. Unmodified polymers used here were as follows: 10 kDa branched PEI (Polysciences, Eppelheim, Germany), 5 kDa branched PEI (a kind gift from BASF, Ludwigshafen, Germany), 5 kDa and 10 kDa linear PEIs (Sigma-Aldrich, Taufkirchen, Germany) and PPI dendrimer generation 4 (SyMO-Chem, Eindhoven, The Netherlands). *N*-Boc-tyrosine-OH, *N*-hydroxysuccinimide, EDC·HCl and benzotriazole-1-yl-oxy-tris-pyrrolidino-phosphonium hexafluorophosphate (PyBOP) were from Carbolution Chemicals (Saarbrücken, Germany). Dry *N*,*N*-Dimethylformamide (DMF) and dimethylsulfoxide (DMSO) was from VWR (Darmstadt, Germany), Trifluoroacetic acid (TFA) and diisopropyalamine (DIPEA) were from Carl Roth (Karlsruhe, Germany). Dialysis tubes (MWCO 1 kDa and 3.5 kDa) were Spectra/Por, from Serva (Heidelberg, Germany).

Cell culture plastics and consumables were purchased from Sarstedt (Nümbrecht, Germany). Cell culture media were from Sigma-Aldrich (Taufkirchen, Germany) and fetal calf serum was from Biochrom (Berlin, Germany). The cell lines HT29 (colorectal carcinoma), PC3 (prostate carcinoma), MV4-11 (biphenotypic B-myelomonocytic leukemia) were obtained from ATCC/LGC Promochem (Wesel, Germany). The cells were routinely tested for Mycoplasma using the Venor^(R)^ GeM Classic kit (Minerva Biolabs, Berlin, Germany) and determined to be free of any contamination. All cell lines were cultivated in a humid atmosphere at 37 °C and 5% CO_2_. HT29 and PC3 cells were grown in Iscove’s Modified Dulbecco Medium (IMDM), and MV-4-11 cells were cultured in RPMI 1640 medium. All media were supplemented with 10% FCS and 2 mM alanyl-glutamine. Cell culture and all transfection experiments were conducted in the absence of antibiotics. Stably dual expressing EGFP/Luciferase-expressing reporter cell lines were prepared by lentiviral transduction as previously described [52].

SiRNA sequences and RT-qPCR primer sequences are given in Table S1.

### 2.2. Chemical Synthesis of Tyrosine-Modified PPI

For the tyrosine-modification of the PPI G4 dendrimer, Boc (*tert*-butyloxycarbonyl) protected tyrosine (0.282 g, 1.0 mmol) was dissolved in 3 mL dry DMSO in a glass vial and PyBOP (0.616 g, 1.18 mmol) was slowly added and stirred for 15 min. In a separate glass vial, the PPI G4 dendrimer (0.1 g, 0.9 mmol in primary amines) was dissolved in dry DMSO and DIPEA (200 µL, 0.151 mol) was added. Next, the pre-activated tyrosine mixture was slowly added to the PPI solution and stirred for 48 h at room temperature. Thereafter, DMSO and other low molecular weight impurities were removed by dialysis (MWCO 1 kDa) against methanol for 6 h. The methanol was removed *in vacuo* and the modified dendrimer was dissolved in 5 mL TFA and left stirring overnight to remove the Boc group. Excess TFA was then removed by co-evaporation with methanol. Finally, the crude polymer was dissolved in 0.1 M HCl and excessively purified by dialysis against 0.05 M HCl for 24 h, then against water for 48 h with intermediate water exchange. Lyophilization yielded the tyrosine-modified PPI as white/yellowish fluffy powder. The degree of substitution was confirmed by ^1^H-NMR (Avance III, 400 MHz, Bruker BioSpin, Rheinstetten, Germany) and calculated as described in [35], indicating an almost complete tyrosine functionalization of the outer primary amines. ^1^H-NMR (400 MHz, D_2_O) δ 1.47–2.17 (m, PPI, 4.023 H), 2.45–3.37 (m, PPI +CH_2_ tyrosine, 9.38 H), 3.93 (m, CH tyrosine, 1 H), 6.86–6.88 (d, H_Ar_ tyrosine, 2H), 7.11–7.13 (d, H_Ar_ tyrosine, 2H).

The tyrosine-modified 5 kDa and 10 kDa branched PEIs (P5Y, P10Y) and linear PEIs (LP5Y, LP10Y) were prepared as described previously according to similar protocols [50,54].

### 2.3. Complex Preparation and Characterization

For standard transfection studies, the cells were seeded at a density of 35,000 cells per well of a 24 well plate in 0.5 mL fully supplemented medium. The following day, the cells were transfected with the polyplexes. The polyplexes were prepared at a polymer/siRNA mass ratio of 2.5 unless indicated otherwise. For a 24 well plate format, 0.4 µg siRNA in 12.5 µL HN buffer (150 mM NaCl, 10 mM HEPES, pH 7.4) and 1 µg tyrosine-modified polymer in 12.5 µL HN buffer were diluted in two separate tubes. The polymer dilution was added to the siRNA dilution, thoroughly mixed and incubated at room temperature for 30 min. After adding the polyplexes to the cells, no further medium change was performed.

The hydrodynamic diameters and zeta potentials of the PPI-G4-Y/siRNA complexes were measured by dynamic light scattering (DLS) and phase analysis light scattering (PALS), using the Brookhaven ZetaPALS system (Brookhaven Instruments, Holtsville, NY, USA). Polyplexes containing 5 µg siRNA in 250 µL total volume were prepared as described above and diluted to 1.7 mL in ultrapure water. The data were analyzed using the manufacturer’s software, applying the viscosity and refractive index of pure water at 25 °C. For size determination, polyplexes were measured in five runs, with a run duration of 1 min per experiment. Zeta potentials were analyzed in ten runs, with each run containing ten cycles using the Smoluchowski model.

To study the complexation efficacy, agarose gel electrophoresis was used. Briefly, 0.2 µg siRNA was complexed in a total volume of 25 µL as described above at the different polymer/siRNA mass ratios indicated in the figure. The polyplexes were mixed with 10× loading dye and run on a 2% (w/v) agarose gel containing ethidium bromide at 80 V in TAE running buffer (20 mM EDTA, 40 mM Tris, 20 mM acetic acid). Unbound siRNA bands were visualized under UV illumination.

For assessing the complex stability of PPI-G4-Y/siRNA complexes, mass ratio of 3.75 was used. Complexes containing 0.2 µg siRNA in 25 µL were incubated with increasing amounts of heparin, incubated for 30 min at room temperature and subjected to agarose gel electrophoresis as described above.

To analyze the influence of FCS on the knockdown efficacies, PPI-G4-Y/siRNA complexes were further incubated in the presence of various FCS concentrations and storage conditions (fresh: 1 h at RT, 3 d at RT or 3 d at 37 °C).

### 2.4. Luciferase Assay and Flow Cytometry

Knockdown efficiencies were analyzed by measuring reporter gene activities (luciferase or enhanced green fluorescent protein, EGFP) 72 h after transfection. For the determination of luciferase activity, the medium was aspirated and the cells were lysed with 300 µL Luciferase Cell Culture Lysis Reagent (Promega, Mannheim, Germany) for 30 min at room temperature. In a test tube 10 µL cell lysate was mixed with 25 µL luciferin reagent (Beetle-Juice Kit, PJK, Kleinblittersdorf, Germany) and immediately measured in a luminometer (Berthold, Bad Wildbad, Germany).

EGFP expression levels were determined by flow cytometry. The cells were harvested by trypsinization and centrifuged for 3 min at 3,000 rpm, prior to resuspension of the cell pellets in FACS buffer (0.5 mL PBS, 1% FCS, 0.1% NaN_3_). 20,000 cells in the vital gate were measured in an Attune^®^ Acoustic Focusing Cytometer (Applied Biosystems, Foster City, CA, USA).

### 2.5. RT-qPCR

Knockdown efficiencies on the mRNA level were analyzed by RT-qPCR. Seventy-two hours after transfection, the total RNA was isolated using a combined TRI reagent (TRIfast, VWR, Darmstadt, Germany) and silica column protocol as previously described [52]. One microgram of total RNA was reverse transcribed using the RevertAid™ H Minus First Strand cDNA Synthesis Kit (Thermo Fisher Scientific; Schwerte, Germany). The cDNA synthesis mixture was incubated at 25 °C for 10 min, 42 °C for 60 min, and heat denatured at 70 °C for 10 min.

For quantitative real time PCR, the cDNA was diluted 1:10 with diethyl pyrocarbonate (DEPC)-water and 4 µL were mixed with 5 µL PerfeCTa SYBR Green FastMix (Quantabio, Beverly, MA) and 1 µL 5 µM primer mix. The RT-qPCR was performed using a Real-Time PCR System (Applied Biosystems) with the following instrument settings: pre-incubation at 95 °C for 2 min, followed by 45 amplification cycles (95 °C for 15 s, 55 °C for 15 s, 72 °C for 15 s). Levels were normalized for RPLP0. Primer sequences are given in Table S2.

### 2.6. Proliferation and LDH Release

Acute cell damage upon transfection was determined by measuring the lactate dehydrogenase (LDH) release, using the Cytotoxicity Detection Kit from Roche (Mannheim, Germany) according to the manufacturer’s protocol. In brief, 35,000 cells were seeded in a 24 well plate. One hour prior to transfection, the medium was replaced with fresh medium and the cells were transfected with the polyplexes as described above containing 0.4 µg siRNA. After 24 h, the medium was harvested. Medium from untreated cells served as negative control and medium of cells treated with Triton X-100 (2% final concentration) was used as positive control (= 100% value) and fresh medium was included for the background value. In a 96 well plate, 50 µL medium was mixed with 50 µL reagent mix and incubated for 30 min in the dark at room temperature. The absorbance was measured at 490 nm and 620 nm as a reference filter in a plate reader (Thermo Fisher, Schwerte, Germany). Acute cytotoxicity values are presented as a percentage of the positive control after subtracting the background.

For the determination of the cell viability/metabolic activity 10,000 cells in 100 µL medium were seeded per well of a 96 well plate. The following day, the cells were transfected with polyplexes at different amounts as indicated in the figure. Numbers of viable/metabolically active cells were measured 72 h after transfection by using the colorimetric WST-8 Cell Counting Kit (Dojindo Molecular Technologies EU, Munich, Germany). Briefly, after replacing the medium with 50 µL of a 1:10 dilution of WST-8 in serum-free medium, the cells were incubated for 1 h at 37 °C. The absorbance was measured at 450 nm and 620 nm as a reference wavelength in a plate reader.

### 2.7. Erythrocyte Aggregation and Hemolysis

Erythrocyte aggregation and hemolysis assays were performed for analyzing possible damaging effects of the polyplexes after incubation. Red blood cells from healthy mice were isolated by repeated washing steps with Ringer’s solution and centrifugation for 5 min at 5,000 rpm until the solution became clear. After the last centrifugation step, the red blood cells were resuspended in 0.9% NaCl solution. For the determination of erythrocyte aggregation, 1 × 10^6^ cells were diluted in 150 µL 0.9% NaCl solution and incubated with the PPI-G4-Y/siRNA polyplexes or with 750 kDa branched PEI as positive control for 2 h at 37 °C. The cells were mounted onto coverslips and examined by bright field microscopy.

The hemolytic activity of the polyplexes was determined by incubating 50 µL polyplex solution with 50 µL cell suspension containing 1.5 × 10^7^ cells in saline for 1 h at 37 °C. Erythrocytes incubated with pure HN buffer served as negative control while cells lysed with 2% Triton X-100 were used as positive control (= 100% value).

### 2.8. Blood Serum Markers and Immunostimulation

Animal studies were performed according to the national regulations and approved by the local authorities (Landesdirektion Sachsen). The mice were kept in cages with rodent chow (ssniff, Soest, Germany) and water available *ad libitum*. Immunocompromised nude mice were maintained and worked with under sterile aseptic conditions.

For analyzing the immunostimulating cytokines TNF-α and INF-γ, PPI-G4-Y/siCtrl complexes containing 10 µg siRNA in 150 µL were intravenous (i.v.) injected twice within 24 h into healthy C57BL/6 mice. The blood was collected four hours after the last injection. Untreated mice served as negative control and mice treated with a single dose of 50 µg lipopolysaccharide (LPS from E.coli O111:B4; Sigma-Aldrich) were used as positive control. The serum levels of TNF-α and INF-γ were measured using ELISA kits (PreproTech, Hamburg, Germany) according the manufacturer’s instructions.

For measuring the blood serum markers alanine-aminotransferase (ALAT), creatinine and urea, healthy nude mice were intraperitoneal (i.p.) and i.v. injected with complexes containing 10 µg siRNA as detailed in the figure. The mice were treated twice within 24 h and the blood was collected 72 h after the first injection. Untreated mice served as negative control. The serum was diluted 1:1 and analyzed using an AU480 (Beckman Coulter, Krefeld Germany).

### 2.9. Statistics

Statistical analyses were performed by Student’s t-test or One-way ANOVA, and significance levels are * = *p* < 0.03, ** = *p* < 0.01, *** = *p* < 0.001 and # = not significant. Unless indicated otherwise, differences between specific and non-specific treatment were analysed, with at least n = 3.

## 3. Results

### 3.1. Generation and Analysis of Tyrosine-Modified PPI-G4 and (L)PEIs, and Their Corresponding siRNA Complexes

Tyrosine-modified (“Y”) fourth generation (“G4”) polypropylenimine (“PPI”) dendrimers (PPI-G4-Y; Figure 1A) were generated according to the synthesis scheme shown in Figure S1A. Based on our previous studies for the tyrosine-modified branched PEIs, all 32 primary amines of the PPI dendrimer were subjected to tyrosine modification. More specifically, the primary amines of the PPI dendrimer were coupled with tyrosine by using PyBOP and DMSO as solvent. The Boc deprotection with TFA and extensive purification by dialysis finally yielded the modified PPI dendrimer as white, fluffy powders. 1H-NMR analysis confirmed the structure of PPI-G4-Y (Figure S1B) and the average number of tyrosine per dendrimer was calculated to be 30. Tyrosine-modified branched and linear PEIs (Figure 1A, lower panels) were prepared as described previously [50,54].

The analysis of complexation efficacies at various polymer/siRNA stoichiometries by agarose gel electrophoresis revealed complete siRNA complexation already at a PPI-G4-Y/siRNA mass ratio of 2.5, as indicated by the absence of the free siRNA band (Figure 1B). Thus, mass ratio 2.5 was used in the further experiments, unless indicated otherwise. The finding of a particularly high complexation efficacy of the tyrosine-modified PPI dendrimer was comparable to previous results on linear or branched low molecular weight PEIs upon their tyrosine-modification, with similarly low mass ratios being sufficient even for the complexation of small RNA molecules like siRNAs. Likewise, PPI-G4-Y/siRNA complexes showed very high stability against heparin displacement, with no siRNA release even at heparin concentrations as high as 60 IU/0.2 µg siRNA (Figure 1C). While similar increases in complex stability had been obtained previously upon tyrosine-modification of PEIs, the complete absence of siRNA release upon heparin treatment as seen here for PPI-G4-Y/siRNA complexes was reminiscent to LP10Y/siRNA complexes [54]. Considering, however, that LP10Y/siRNA- as well as PPI-G4-Y/siRNA complexes display biological activity which must be based on the release of intact siRNA molecules, this demonstrates that heparin displacement seems to somewhat over-estimate complex stabilities and thus only poorly reflect the situation in biological media. Still, it should be noted that PPI-G4-Y/siRNA complexes, comparable to LP10Y/siRNA complexes, show particularly high stabilities even when prepared at very low polymer/siRNA ratios.

Zetasizer measurements revealed rather large PPI-G4-Y/siRNA complex sizes with diameters of almost 600 nm already at mass ratio 2.5. They increased even further when using more polymer for complexation and were then in the range of ~ 800–900 nm (Figure 1D). Thus, despite essentially complete siRNA complexation already at mass ratio 2.5 (see above), the addition of more PPI-G4-Y dendrimer still contributed to the complex formation. This was also associated with alterations in the zeta potential. More specifically, while PPI-G4-Y/siRNA complexes were slightly negative at mass ratio 2.5 and, to a lesser extent, at mass ratio 3.75, they turned even slightly positive at the higher mass ratio 5 (Figure 1D). In conclusion, comparably large complexes with very little surface charge were obtained when using PPI-G4-Y.

### 3.2. Biological Efficacies of siRNA Complexes Based on PPI-G4-Y- or Various Tyrosine-Modified PEIs

**Figure 2:**
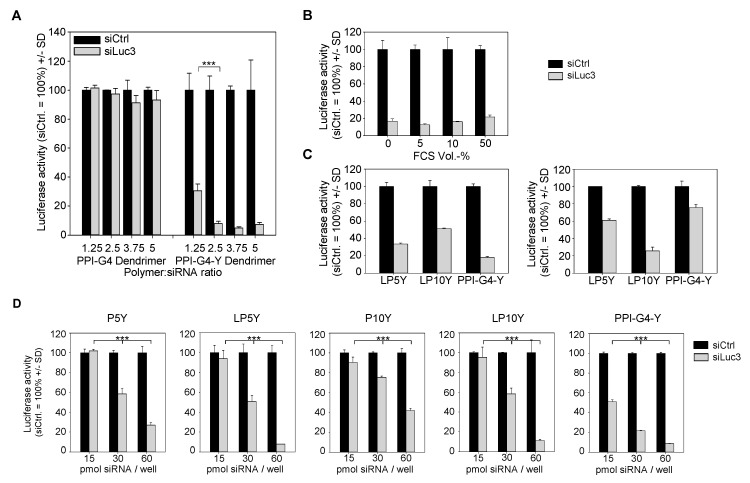
(**A**) Knockdown efficacies of PPI-G4 dendrimer-based siRNA complexes with (right) or without (left) tyrosine modification, as determined by luciferase knockdown in PC3-EGFP/Luc reporter cells. Bars show results upon transfection with complexes containing negative control siRNA (black) or luciferase-specific siRNA (grey), respectively, with black bars normalized to 100%. UT: untreated. (**B**) Knockdown efficacies upon pre-incubation of the complexes in the presence of FCS at various concentrations. (**C**) Knockdown efficacies of tyrosine-modified PPI- or PEI-based siRNA complexes in HT29-EGFP/Luc (upper panel) or MV4-11-EGFP/Luc cells (lower panel). (**D**) Dose-dependent knockdown efficacies of various tyrosine-modified PPI- or PEI-based siRNA complexes in HT29-EGFP/Luc cells.

The beneficial effects of tyrosine-modification also became evident when analyzing knockdown efficacies. The use of PPI-G4 dendrimers for siRNA transfection into stably luciferase expressing PC3-EGFP/Luc reporter cells yielded no reduction of luciferase at all, independent of complex stoichiometries up to mass ratio 5 (Figure 2A, left).

In stark contrast, > 90% reduction of luciferase activity was observed upon incubation of cells with PPI-G4-Y/siRNA complexes. In agreement with the above results, ratio 2.5 proved to be sufficient for reaching maximum knockdown efficacy (Figure 2A, right).

When analyzing luciferase activity values normalized to untreated control cells, rather than those normalized to the respective PPI-G4-Y/negative control siRNA complexes as in Figure 2A, it was also seen that PPI-G4-Y/siRNA complexes prepared at ratios 1.25 or 2.5 exerted very few non-specific effects, with luciferase activity upon PPI-G4-Y/siCtrl transfection remaining comparable with untreated (Figure S2A, right). In contrast, at least in this cell line, non-specific reduction of luciferase activity was observed at ratios 3.75 or higher. Knockdown efficacies were not impaired by high protein content, i.e., no decrease in siRNA activity was seen in the presence of FCS at a concentration as high as 50% (Figure 2B). In fact, higher protein concentrations rather protected knockdown activity upon storage at various temperatures for three days (Figure S3). Gene targeting on the protein level, as determined by reduced luciferase activity, was also paralleled by decreased mRNA levels (Figure S2B), further substantiating the specificity of the siRNA-mediated knockdown. Results were comparable to previously described tyrosine-modified PEIs, e.g., to P5Y/siRNA complexes.

**Figure 3:**
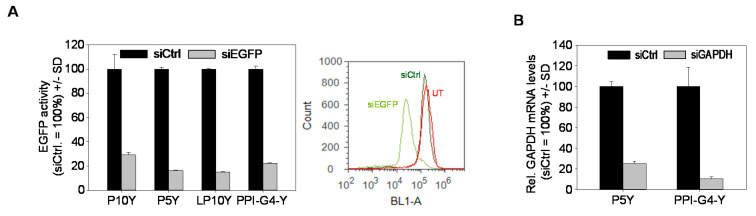
(**A**) Knockdown efficacies of various tyrosine-modified PPI- or PEI-based siRNA complexes targeting EGFP in PC3-EGFP/Luc cells (left). EGFP levels were determined by flow cytometry (see right panel and Figure S2C for original data). (**B**) Reduction of GAPDH mRNA levels in PC3 cells.

Knockdown efficacies and the definition of optimal complexes, however, were also found to be dependent on the cell line. In HT29-EGFP/Luc colon carcinoma cells, PPI-G4-Y/siRNA complexes showed better knockdown activity than their counterparts based on tyrosine-modified linear 5 kDa or 10 kDa PEI (LP5Y/siRNA or LP10Y/siRNA complexes; Figure 2C). In particular the LP10Y/siRNA complexes which had previously been found to efficiently mediate gene knockdown showed only moderate ~ 50% reduction of their target gene (Figure 2C, upper panel). In contrast, in MV4-11-EGFP/Luc B-myelomonocytic leukemia cells the same complexes were particularly efficient while little knockdown was achieved when using PPI-G4-Y (Figure 2C, lower panel). Maximum achievable knockdown efficacies, however, also proved to be dose-dependent. The lesser activity of LP10Y/siRNA complexes in HT29-EGFP/Luc cells observed above was not seen any more when doubling complex amounts: while at 15 and 30 pmol/well major differences between both polymers were seen, 60 pmol led to a very profound > 90% reduction of luciferase also in the case of LP10Y-based complexes (Figure 2D, right). This was comparable with LP5Y/siRNA complexes, whereas their counterparts based on branched 5 or 10 kDa PEI (P5Y, P10Y) showed somewhat lesser efficacy in HT29 cells (Figure 2D, left and center). In other cell lines, knockdown efficacies were rather comparable and were also seen when targeting other genes. In PC3-EGFP/Luc cells, the transfection of siRNAs specific for EGFP led to profound reduction of EGFP fluorescence, as determined by flow cytometry (Figure 3A, Figure S2C).

In the case of all polymers, the direct comparison between untreated and negative control siRNA transfected cells revealed no differences, indicating the absence of non-specific effects. Finally, when switching to an endogenous gene rather than stably, but ectopically expressed reporter genes, similar results were obtained. As shown for glyceraldehyde 3-phosphate dehydrogenase (GAPDH), PPI-G4-Y/siRNA complexes led to an ~ 90% reduction of the target gene (Figure 3B).

Taken together, this identifies complexes based on PPI-G4-Y, as alternative to tyrosine-modified linear or branched PEIs, as very efficient for siRNA-mediated knockdown in various cell lines, with some differences seen dependent on the cell line and the complex amounts used for transfection.

### 3.3. Biocompatibility of siRNA Complexes Based on PPI-G4-Y- or Various Tyrosine-Modified PEIs

**Figure 4:**
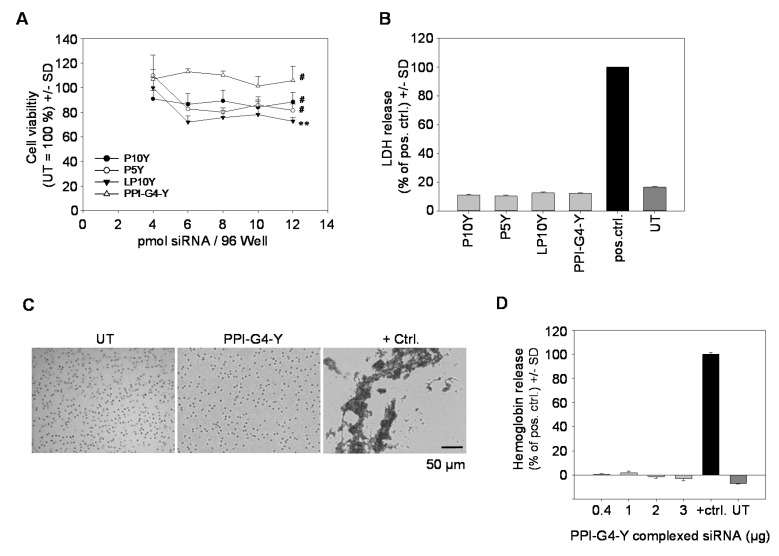
(**A**) Analysis of PC3 cell viability by WST-8 based measurement of viable cells upon transfection with various tyrosine-modified PPI- or PEI-based siRNA complexes at different amounts (indicated by siRNA amounts on the x-axis). Statistics analyze differences of the highest siRNA amounts (12 pmol) to untreated. (**B**) Assessment of acute cytotoxicity in PC3 cells by lactate dehydrogenase (LDH) into the medium. (**C**) Analysis of hemoglobin aggregation and (**D**) of hemoglobin release upon incubation of erythrocytes with PPI-G4-Y/siRNA complexes.

Beyond knockdown efficacy, biocompatibility is a major variable determining optimal complexes for transfection. Complexes based on PPI-G4-Y as well as those based on tyrosine-modified linear or branched PEIs proved to be highly biocompatible, as seen in cell viability assays in PC3 cells (Figure 4A). Notably, in particular transfection with PPI-G4-Y/siRNA complexes preserved cell viability by 100%. The absence of acute toxicity was also confirmed by LDH release assays, indicating no acute cell damage upon transfection with any of the tested complexes (Figure 4B). In line with this, no adverse effects on erythrocytes were observed, as shown here for PPI-G4-Y/ siRNA complexes. Erythrocytes incubated with PPI-G4-Y/siRNA complexes revealed no signs of aggregation, leaving them indistinguishable from their untreated counterparts (Figure 4C). Likewise, independent of complex amounts, no hemoglobine release from erythrocytes was detected (Figure 4D).

### 3.4. In Vivo Biocompatibility of siRNA Complexes Based on PPI-G4-Y- or Various Tyrosine-Modified PEIs

**Figure 5:**
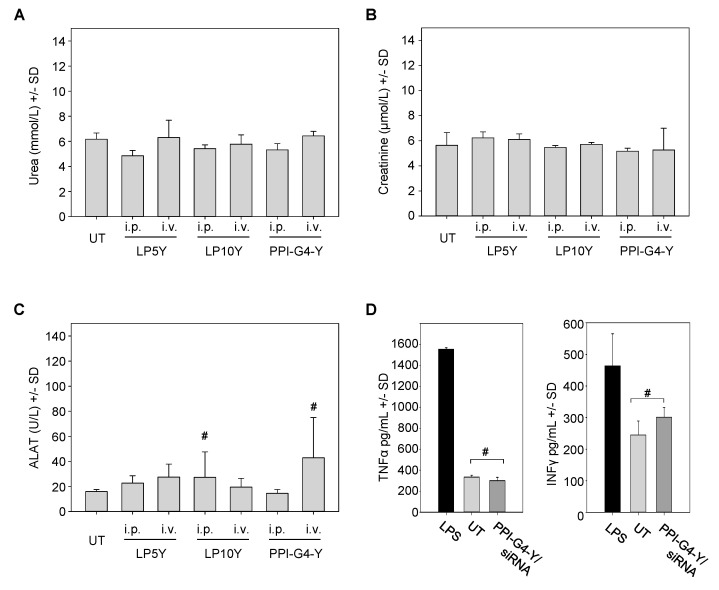
Determination of serum levels of (**A**) urea, (**B**) creatinine and (**C**) alanine-aminotransferase (ALAT) levels upon intraperitoneal (i.p.) or intravenous (i.v.) injection of mice with the tyrosine-modified PPI- or PEI-based siRNA complexes indicated on the x-axis. (**D**) Measurement of TNFα (left) and INFγ serum levels (right) upon i.v. injection of PPI-G4-Y/siRNA complexes.

Studies on the biocompatibility of nanoparticles based on tyrosine-modified PPI or PEIs were also extended towards their application in vivo. Administration routes were intravenous (i.v.) or, considering the previously observed high efficacy and systemic availability of PEI-based nanoparticles, intraperitoneal (i.p.) injection [50,55]. In the case of PEI/siRNA complexes, these two modes of administration had previously shown marked differences in biodistribution and bioavailability, and thus both were included. After two injections, blood samples were taken and analyzed for serum markers. Levels of urea (Figure 5A) and creatinine (Figure 5B) after treatment were found identical to the untreated negative control, indicating the absence of nephrotoxic effects. For the assessment of hepatotoxicity, alanine-aminotransferase levels were determined. In some cases, minor increases in alanine-aminotransferase (ALAT) levels were detected, but did not reach statistical significance (Figure 5C). Finally, immune stimulation was measured as well. In this case, studies were restricted to i.v. administration since cytokine activation occurs in a narrow time window and thus requires immediate bioavailability of the whole sample. To cover a very short as well as a somewhat longer time range, two injections of PPI-G4-Y/siRNA complexes within 24 h were performed, prior to the determination of TNFα and IFNγ levels. While a profound increase in the levels of both cytokines was observed in the lipopolysaccharide (LPS) positive control group, treatment with PPI-G4-Y/siRNA complexes led to no (TNFα) and or a slight, statistically not significant (IFNγ) alteration in serum levels (Figure 5D). In line with this, no alterations in animal appearance or behavior were observed throughout the experiments.

## 4. Discussion

Tyrosine-modified polymers based on linear or branched PEIs have been identified as highly efficient for siRNA delivery in this and previous studies [50,51,52,54,56,57,58]. In this paper, we introduce for the first time the extension of this approach towards PPI dendrimers. In contrast to their non-modified counterparts, the tyrosine-grafted polymers show substantially increased complexation efficacy and complex stability, which is particularly critical for small oligonucleotides like siRNAs and also allows for using smaller polymers at lower mass ratios. The latter aspect may also provide a difference with regard to previous studies on unmodified PPI, where, when using higher siRNA concentrations than in our experiments, considerable knockdown was observed [29,30]. The positive effect of tyrosine grafting may rely on the contribution of others than electrostatic interactions, including π−π and cation−π interactions as suggested previously [59,60,61]. Notably, however, even the markedly enhanced complex stabilities still allow for efficient intracellular siRNA release, as seen from the high knockdown efficacies. In this context, it should be noted that the analysis of complex decomposition by heparin displacement has repeatedly proven to be insufficient since its results, falsely, suggested no siRNA release at all. Rather, studies in the presence of biological media like protein-containing cell or tissue lysates seem to be more appropriate in this regard [54].

Another major bottleneck for nanoparticle activity is their efficient cellular delivery and uptake. Notably, despite their large sizes, especially in the case of the PPI-G4-Y/siRNA complexes, and their very low (if at all) surface charge, cellular internalization of tyrosine-modified PEI- or PPI-based nanoparticles proved to be high. This indicates that surface charge is not a major determinant of nanoparticle activity. Still, the larger sizes may come with issues in vivo, e.g., regarding the penetration into intact tissue. In the case of P10Y/siRNA [50] or LP10Y/siRNA complexes [54], however, high biological activities in xenograft tumors were observed, arguing against major problems related to size. While it remains to be seen if this is also true for the even larger PPI-G4-Y/ siRNA complexes, it can already be noted that even sizes of almost 600 nm did not lead to issues regarding biocompatibility after i.v. injection. Also, limited tissue penetration and thus altered biodistribution may prove beneficial with regard to specificities for certain compartments and cell types. In this context, it could be of interest that we found PPI-G4-Y/siRNA complexes to be particularly efficient for macrophage transfection in vitro (data not shown). Thus, they may well represent candidates for preferentially transfecting these cells also in vivo, without the introduction of targeting moieties for targeted delivery. Taken together, these aspects provide the basis and clearly call for future in vivo experiments, covering biodistribution upon different modes of administration, gene knockdown in various target cells including tumor cells, stroma cells as well as hematopoietic cells, and therapeutic effects in relevant tumor models. While beyond the scope of this paper, we have already selected a relevant siRNA dosage (10 µg) for our toxicity studies presented here, based on previous results on PEI/siRNA or P10Y/siRNA complexes [50,55]. Thus, the combination of these and previous data, i.e., (i) the absence of toxicity at (ii) dosages previously identified in PEI-based systems as relevant for therapy, and (iii) in the light of the even enhanced transfection efficacy seen here, provides a strong basis for extensive in vivo and therapy studies.

The very high biocompatibilities in vitro and in vivo described here for PPI-G4-Y/siRNA complexes as well as in our previous studies for tyrosine-modified PEIs may be readily explained by the low nanoparticle zeta potentials, considering that positive surface charges have been associated with cytotoxicity [62], and the comparably little polymer amounts required for siRNA complexation. Still, more detailed studies will be required for comprehensively analyzing their toxicological profile. As reported previously, G4 PPI induced DNA damages in a COMET assay but no effects were observed in the case of a maltose-modified G4 PPI derivative. Likewise, G4 PPI, but not the maltose-modified G4 PPI, showed toxic effects in vivo, e.g., changes in serum parameters, body weight and behavior. These studies indicate that PPI dendrimers, when chemically modified, are interesting starting materials for synthesizing new effective and biocompatible polymers for nucleic acid delivery. In this regard, the approach of combining tyrosine-grafting with the introduction of biodegradable linkers at defined branching points in the polymer will be of particular interest. It should be noted, however, that the systems reviewed and described here rely on a comparably minor chemical modification, thus increasing the likeliness of their possible translation into the clinics. It must be kept in mind that successful systems for therapeutic siRNA delivery will have to combine maximum efficacy and biocompatibility with favorable properties with regard to manufacturing, upscaling, charge variability and GMP production.

## 5. Conclusions

This paper, as well as previous studies, establish tyrosine-modified PPIs or PEIs as particularly promising polymeric systems for siRNA formulation and delivery. Low chemical complexity, as in the case of tyrosine modifications, and particularly defined structure and size, as in the case of PPI dendrimers, in combination with the optimal molecular weight (G4) may leave PPI-G4-Y as particularly promising for siRNA formulation.

## Supplementary Materials

The following are available online at https://www.mdpi.com/2079-4991/10/9/1809/s1, Figure S1: Synthesis scheme and 1H-NMR analysis of PPI-G4-Y. Figure S2: Knockdown efficacies of various tyrosine-modified PPI- or PEI-based siRNA complexes. Figure S3: Knockdown efficacies upon storage of PPI-G4-Y/siRNA complexes at various temperatures for three days, in the presence of different FCS concentrations.
