# Supplementary material for: Tyrosine-Modification of Polypropylenimine (PPI) and Polyethylenimine (PEI) Strongly Improves Efficacy of siRNA-Mediated Gene Knockdown
Source: Nanomaterials (Basel). 2020 Sep 10;10(9):1809. doi: 10.3390/nano10091809 (PMC7557430; doi:10.3390/nano10091809)
Supplement: Supplementary file 1 [file nanomaterials-10-01809-s001.pdf]

nanomaterials

Supplementary Materials

Tyrosine-Modification of Polypropylenimine (PPI) and Polyethylenimine (PEI) Strongly Improves Efficacy of siRNA-Mediated Gene Knockdown

Sandra Noske, Michael Karimov, Achim Aigner * and Alexander Ewe *

Rudolf-Boehm-Institute for Pharmacology and Toxicology, Clinical Pharmacology, Leipzig University, Faculty of Medicine, Leipzig, 04107, Germany; Sandra.noske@medizin.uni-leipzig.de (S.N.); michael.karimov@medizin.uni-leipzig.de (M.K.)

* Correspondence: achim.aigner@medizin.uni-leipzig.de (A.A.); alexander.ewe@medizin.uni-leipzig.de (A.E.); Tel.: +49-(0)341-9724660 (A.A.)

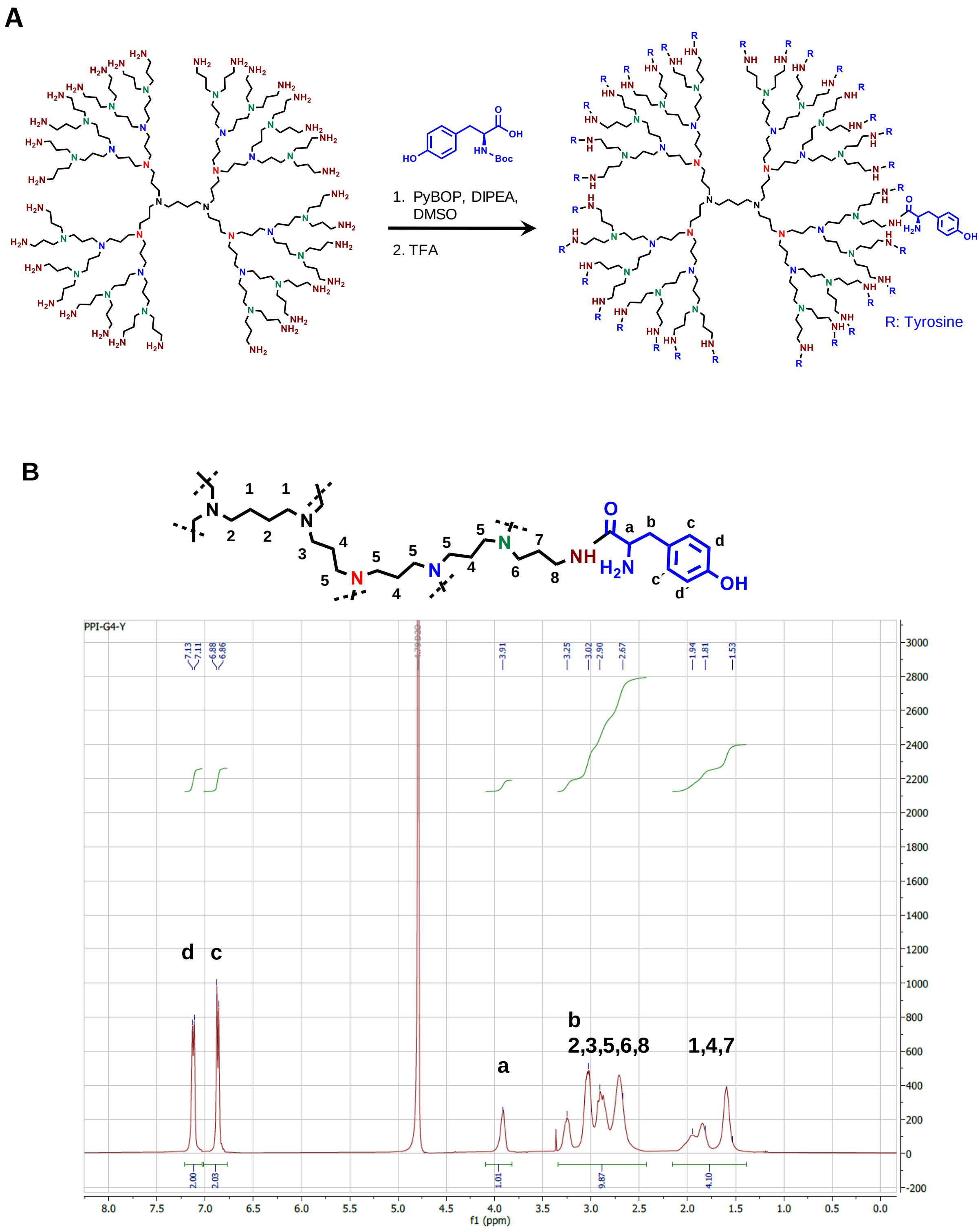

Figure S1. (**A**) Synthesis scheme for the generation of PPI-G4-Y. (**B**) 1H-NMR analysis of PPI-G4-Y. Relevant signals are highlighted.

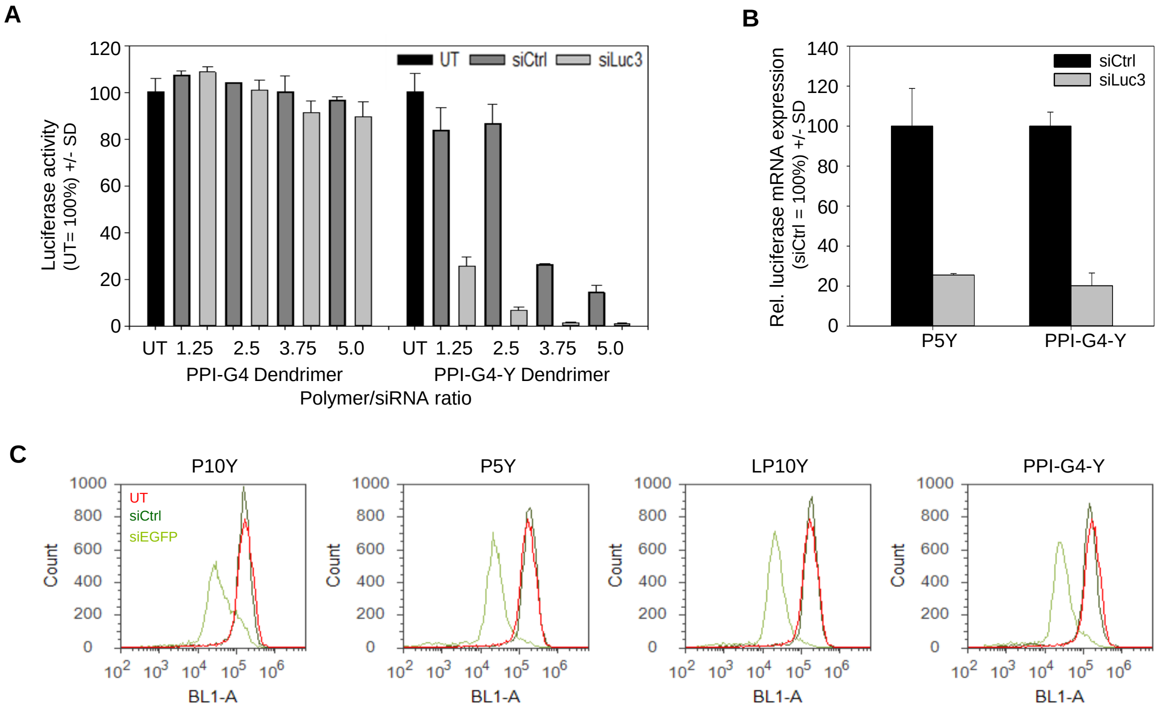

Figure S2. (**A**) Knockdown efficacies of PPI-G4 dendrimer-based siRNA complexes with (right) or without (left) tyrosine modification, as determined by luciferase knockdown in PC3-EGFP/Luc reporter cells. Bars show results upon transfection with complexes containing negative control siRNA (black) or luciferase-specific siRNA (grey), respectively, with bars normalized for untreated (UT) negative control. (**B**) Luciferase knockdown on the mRNA level in PC3-EGFP/Luc cells, as determined by RT-qPCR. (**C**) Knockdown efficacies of various tyrosine-modified PPI- or PEI-based siRNA complexes targeting EGFP in PC3-EGFP/Luc cells (original flow cytometry data from Figure 3A).

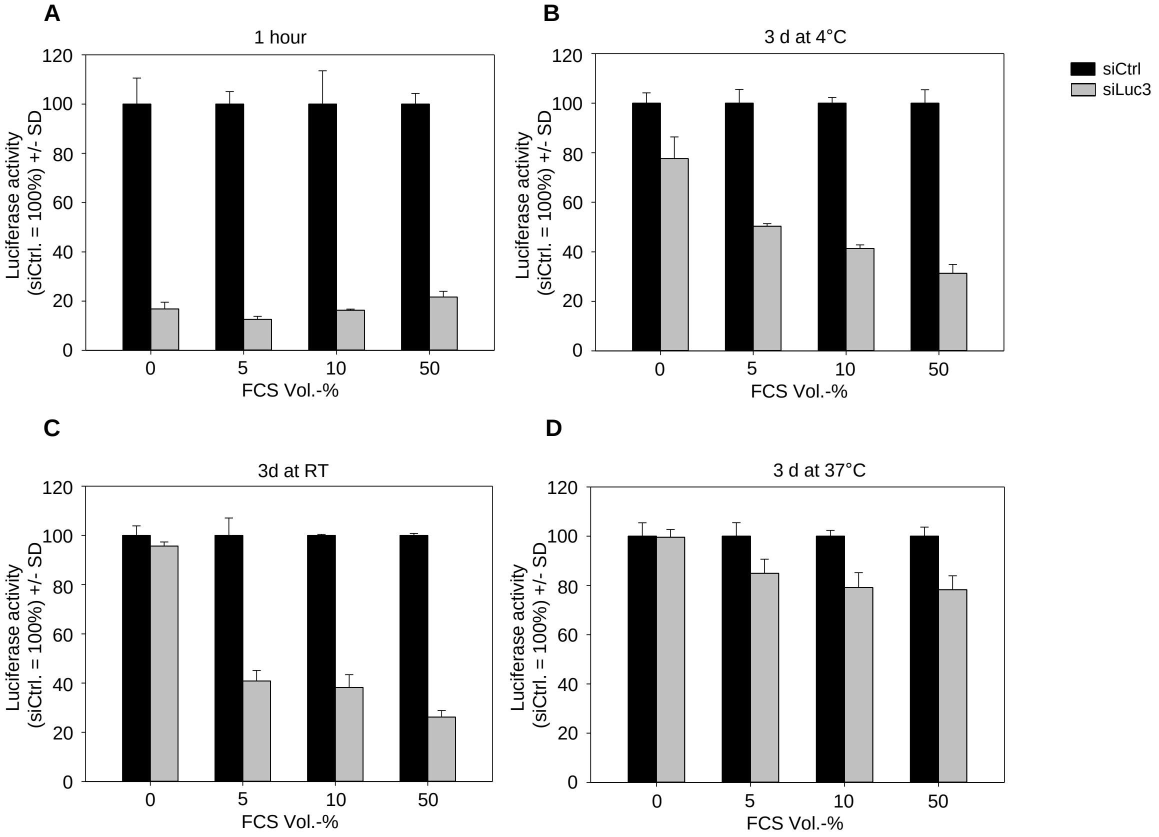

Figure S3. Knockdown efficacies of PPI-G4 dendrimer-based siRNA complexes upon storage for three days at different temperatures, in the absence or presence of FCS at the indicated concentrations, as determined by luciferase activity in PC3-EGFP/Luc cells.

Table S1. siRNAs used in this study.

**siRNA**		**Sequence (5′ – 3′)**	**Vendor**
siLuc 2	*sense*	CGUACGCGGAAUACUUCGA dTdT	Dharmacon Lafayette, CO, USA
	antisense	UCGAAGUAUUCCGCGUACG dTdT	
siLuc 3	*sense*	CUUACGCUGAGUACUUCGA dTdT	Eurogentec, Seraing, Belgium
	antisense	UCGAAGUACUCAGCGUAAG dTdT	
siGAPDH	*sense*	CCUCAACUACAUGGUUUAC dTdT	Eurogentec Seraing, Belgium
	antisense	GUAAACCAUGUAGUUGAGG dTdT	
siEGFP	*sense*	GCAGCACGACUUCUUCAAG dTdT	Eurogentec Seraing, Belgium
	antisense	CUUGAAGAAGUCGUGCUGC dTdT	

Table S2. Sequences of primers used in this study.

**Primer**		**Sequence (5′ – 3′)**	**Vendor**
RPLP0	forward	TCTACAACCCTGAAGTGCTTGAT	Eurofins Genomics, Ebersberg, Germany
	reverse	CAATCTGCAGACAGACACTGG	
GAPDH	forward	GGTGTGAACCATGAGAAGTATGA	Bianoscience, Gera, Germany
	reverse	GAGTCCTTCCACGATACCAAAG	
Luc3	forward	TTACACCCGAGGGGGATGAT	Eurofins Genomics, Ebersberg, Germany
	reverse	TCTCACACACAGTTCGCCTC	
